# Factors associated with the use of complementary and alternative medicines for prostate cancer by long-term survivors

**DOI:** 10.1371/journal.pone.0193686

**Published:** 2018-03-07

**Authors:** Sam Egger, Suzanne Hughes, David P. Smith, Suzanne Chambers, Clare Kahn, Annette Moxey, Dianne L. O’Connell

**Affiliations:** 1 Cancer Research Division, Cancer Council New South Wales, Sydney, New South Wales, Australia; 2 School of Public Health, University of Sydney, Sydney, New South Wales, Australia; 3 Menzies Health Institute, Queensland, Griffith University, Gold Coast, Queensland, Australia; 4 Cancer Research Centre, Cancer Council Queensland, Brisbane, Queensland, Australia; 5 Prostate Cancer Foundation of Australia, Sydney, New South Wales, Australia; 6 Exercise Medicine Research Institute, Edith Cowan University, Perth, Western Australia, Australia; 7 Institute for Resilient Regions, University of Southern Queensland, Toowoomba, Queensland, Australia; 8 School of Medicine and Public Health, University of Newcastle, Callaghan, New South Wales, Australia; Texas Technical University Health Sciences Center, UNITED STATES

## Abstract

**Objective:**

To assess whether the use of complementary and alternative medicines therapies (CAMs) for prostate cancer and/or its treatment side effects by long-term survivors is associated with selected socio-demographic, clinical, health-related quality-of-life (HRQOL) and/or psychological factors.

**Design, setting and participants:**

The Prostate Cancer Care and Outcomes Study (PCOS) is a population-based cohort study of men with prostate cancer who were aged less than 70 years at diagnosis in New South Wales, Australia. Included in these analyses were men who returned a 10-year follow-up questionnaire, which included questions about CAM use.

**Methods:**

Validated instruments assessed patient’s HRQOL and psychological well-being. Poisson regression with robust variance estimation was used to estimate the adjusted relative risks of current CAM use for prostate cancer according to socio-demographic, clinical, HRQOL and psychological factors.

**Results:**

996 of 1634 (61%) living PCOS participants completed the 10-year questionnaire. Of these 996 men, 168 (17%) were using CAMs for prostate cancer and 525 (53%) were using CAMs for any reason (including prostate cancer). Those using CAM for prostate cancer were more likely to be regular or occasional support group participants (vs. no participation RR = 2.02; 95%CI 1.41–2.88), born in another country (vs. Australian born RR = 1.59; 95%CI 1.17–2.16), have received androgen deprivation treatment (ADT) since diagnosis (RR = 1.60; 95%CI 1.12–2.28) or in the past two years (RR = 2.34; 95%CI 1.56–3.52). CAM use was associated with greater fear of recurrence (RR = 1.29; 95%CI 1.12–1.48), cancer-specific distress (RR = 1.15; 95%CI 1.01–1.30), cancer-specific hyperarousal (RR = 1.17; 95%CI 1.04–1.31), cancer locus of control (RR = 1.16; 95%CI 1.01–1.34) and less satisfaction with medical treatments (RR = 0.86; 95%CI 0.76–0.97), but not with intrusive thinking, cognitive avoidance, depression, anxiety or any HRQOL domains.

**Conclusions:**

In this study, about one in six long term prostate cancer survivors used CAMs for their prostate cancer with use centred around ADT, country of birth, distress, cancer control, fear of recurrence and active help seeking.

## Introduction

The use of complementary and alternative medicines (CAMs) by cancer patients remains an emotionally charged issue. Critics of these therapies often cite the paucity of scientific evidence justifying their use, their potential for adverse effects, their potential for interaction with conventional treatments, the possibility that patients might use them as alternatives to conventional treatments and the potential for financial exploitation of vulnerable people [1–3]. Advocates, on the other hand, often point to the limited evidence supporting the use of some conventional medicines [4], while others argue that CAMs should not be held to the same standards of proof as evidence-based medicines [5]. Regardless of the arguments for and against, CAM use by cancer patients is a worldwide phenomenon [1] that is likely to grow as cancer incidence and survival increase. Consequently, it is important for clinicians to be aware of the types of CAMs that cancer patients are likely to use and the factors that motivate their use, particularly for patients with cancer types known to be associated with this use.

The National Cancer Institute’s Office of Cancer Complementary and Alternative Medicines (OCCAM) defines ‘complementary and alternative medicine’ as “any medical system, practice, or product that is not thought of as standard care” [6]. According to the OCCAM, broad categories of CAMs include alternative medical systems, energy therapies, exercise therapies, manipulative and body-based methods, mind-body interventions, nutritional therapeutics, pharmacologic and biologic treatments and spiritual therapies. Previous research has shown that CAM use is common amongst prostate cancer patients. A 2011 systematic review reported that in 39 studies, the median prevalence of CAM use following a diagnosis of prostate cancer was 30% [7]. Previous studies have also identified various predictors of CAM use amongst prostate cancer patients such as higher education [8,9] and more advanced or aggressive cancer at diagnosis [10,11]. To our knowledge, however, there have been no studies that have examined factors associated with CAM use for prostate cancer exclusively among long-term survivors. This is despite the fact that substantial numbers of men are living with a diagnosis of prostate cancer and the adverse consequences that can follow for many years after diagnosis [12]. In Australia, for example, prostate cancer is expected to remain the most commonly diagnosed non-cutaneous cancer among males in 2017, accounting for 12% of all reportable cancers [13]. With five-year relative survival of around 95% [13], and nearly 100% for those diagnosed with localised disease [14], there will be a significant and increasing number of Australian men living with a previous diagnosis of prostate cancer.

In this cross-sectional analysis, we used information from a group of long-term survivors of prostate cancer (mean = 10 years) from Australia’s most populous state, New South Wales (NSW), to examine whether ‘current CAM use for prostate cancer and/or its treatment side effects’ (hereafter abbreviated as ‘CAM use for prostate cancer’) is associated with selected socio-demographic, clinical, health-related quality-of-life (HRQOL) and/or psychological factors.

## Materials and methods

### Study sample

The Prostate Cancer Care and Outcomes Study (PCOS) is a population-wide longitudinal cohort study conducted in NSW, Australia, with a primary objective of assessing the effects of various treatments on HRQOL after diagnosis of prostate cancer. A total of 3195 men aged less than 70 years with histopathologically confirmed T1-4 prostate cancer diagnosed between October 2000 and October 2002 were identified through the NSW Cancer Registry and after consent had been given by their doctor, were invited to participate in PCOS. Of these 1995 completed a baseline interview (S1 Fig). As of January 2011, 1634 men were still alive of whom 1427 remained in PCOS and were invited to participate in a 10-year follow-up survey (mean of 10 years after diagnosis; range 9–12 years) assessing their use of CAMs and various HRQOL and psychological domains. Of the 1634 men, 996 (61%) completed and returned the 10-year questionnaire. Additional details of the initial recruitment process for PCOS are provided elsewhere [15,16]. PCOS was approved by the human research ethics committees of the Cancer Council NSW, the Cancer Institute NSW, and the NSW Department of Health. The 10-year follow-up survey was approved by the Cancer Council NSW Human Research Ethics Committee (Approval number: 2010#244).

### Data collection

#### Clinical and socio-demographic data

Baseline clinical data were collected for each participant by either a trained field worker or the treating doctor using a data collection form and protocol. These data were collected between 12 and 24 months after the histological diagnosis of prostate cancer and included prostate-specific antigen (PSA) level at diagnosis, Gleason score and clinical stage at diagnosis, and treatment received within 12 months of diagnosis. Place and socio-economic status of the man’s place of residence at diagnosis were based on the Accessibility/Remoteness Index of Australia (ARIA+) [17] and the Socio-Economic Indexes for Areas (SEIFA) [18] respectively. Highest level of education completed was self-reported in the baseline PCOS survey. Information on prostate cancer treatments received was obtained from the treating doctor’s records (diagnosis to 12 months) and from linked administrative health datasets (diagnosis to current follow-up). For each man who consented, treatment data were obtained from Medicare Australia and NSW Health’s Admitted Patient Data Collection [19]. Clinical and socio-demographic information obtained in the 10-year survey included current place of residence, employment status, marital status, support group participation and the man’s knowledge of whether the cancer has spread.

#### Psychological and health-related quality-of-life domains

A number of previously validated psychological and HRQOL instruments were included in the 10-year survey including: a 6-item course of cancer subscale from the Cancer Locus of Control scale measuring each man’s perceived control of the course of their cancer [20]; Kornblith's 5-item Cancer Fear of Recurrence scale [21]; the 22-item Impact of Event Scale-Revised (IES-R) [22] measuring distress, hyperarousal, intrusive thinking and cognitive avoidance associated with having prostate cancer; the14-item Hospital Anxiety and Depression Scale (HADS) [23] measuring anxiety and depression; the 26-item Expanded Prostate cancer Index Composite Short Form (EPIC-26) [24] measuring urinary incontinence, urinary irritative/obstructive, bowel, sexual and hormonal summary scores, and also measuring urinary, bowel and sexual bother scores from a subset of questions common to both the University of California-Los Angeles Prostate Cancer Index (UCLA-PCI) [25] and the EPIC-26; and the 12-item Short Form-12 (SF-12) [26] scale measuring the mental and physical dimensions of HRQOL. Satisfaction with medical treatments was measured on a 1-item 5-point Likert scale from the Expanded Prostate cancer Index Composite Long Form (EPIC-50) [27]. For each psychological domain, higher scores indicate higher levels of the psychological attribute. For each HRQOL domain, higher scores indicate better HRQOL (which corresponds to less bother for the bother domains).

#### Complementary and alternative medicines

Questions relating to CAM use were broadly based on the I-CAM-Q [28], but with modifications to address issues specific to prostate cancer survivors. While the I-CAM-Q has been a widely used instrument for measuring CAM use, it is yet to be fully evaluated. CAMs comprised dietary supplements, non-specialist treatments and self-help therapies. Dietary supplements included vitamins, minerals and herbal medicines. Non-specialist treatments were defined as treatments provided by practitioners other than a medical specialist such as acupuncture, biofeedback and homeopathy. Self-help therapies were defined as therapies that the survivor could do themselves such as relaxation techniques, meditation, yoga or prayer. For each category of CAM, men were asked about their current and previous use of those therapies for prostate cancer, and their current use of those therapies for other health conditions or general well-being.

### Statistical methods

Poisson regression with robust variance estimation [29] was used to estimate the adjusted relative risks of current CAM use for prostate cancer according to socio-demographic characteristics, clinical characteristics, psychological and HRQOL domains. The dependent variable in all regression models was CAM use for prostate cancer (yes or no). Independent variables included age (<65, 65–69, 70–74, 75+ years; age ranged from 52 to 80 years), education (university or college degree, high school, less than high school), socio-economic status of place of residence (divided into quintiles using SEIFA), place of residence (major city, inner regional, outer regional/ remote/ very remote based on ARIA+), health insurance (private health insurance- with extras, private health insurance- without extras, Medicare only), employment status (in full time paid work, in part time paid work, retired/unemployed, self-employed), married or in defacto partner relationship (no, yes), participation in a support group (no contact with support groups, receive newsletter only, participate regularly or occasionally), country of birth (Australia, elsewhere), overall cancer severity at diagnosis (localised low risk, localised intermediate risk, localised high risk, stage T3-4) [30] and treatments used since diagnosis (active surveillance/watchful waiting (AS/WW), prostatectomy, external beam radiotherapy (EBRT)/ brachytherapy, bone EBRT, androgen deprivation treatment (ADT), chemotherapy/bisphosphonates). Other independent variables included PSA level at diagnosis (<4, 4 to <10, 10 to <20, 20+ng/mL), Gleason score at diagnosis (<7, 7, >7), clinical stage at diagnosis (T1a-c, T2a-c, T3a-c/T4a), knowledge of cancer spread (no, yes) and prostate cancer treatments used in the past two years. In order to avoid collinearity, however, these other variables were not included in models simultaneously with overall cancer severity at diagnosis and treatments used since diagnosis. Tests for linear associations between CAM use and socio-demographic and clinical characteristics were performed by substituting the nominal versions of socio-demographic and/or clinical variables with continuous or ordinal versions where appropriate. For ordinal variables that were not interval scaled (eg. health insurance), consecutive integers were used for coding when testing for linear associations. Psychological and HRQOL variables were included one at a time as linear continuous independent variables in regression models after standardising each variable to have variance equal to one. Hence, relative risks for psychological and HRQOL variables indicate the change in the probability/risk of CAM use per standard deviation increase in the variable [31]. Subjects with missing data for any psychological or HRQOL domain were excluded from analyses relating to that particular domain.

## Results

Of the 996 men who returned a 10-year follow-up questionnaire, 168 (16.9%) were current users of CAMs for prostate cancer and 525 (52.7%) were current users of CAMs for any reason (including prostate cancer) (Table 1). The most common categories of CAMs used for prostate cancer were dietary supplements (n = 115, 11.6%), and self-help activities (n = 73, 7.3%). The reasons for and sources of information on CAM use for prostate cancer are shown in S1 Table.

**Table 1:** Categories and subcategories of CAMs currently used for prostate cancer and/or treatment side effects and for any reason.

CAMs currently used ^	For prostate cancer and/or treatment side effects n (% of 996)	For any reason n (% of 996)
**Any CAM**:	**168 (16.9)**	**525 (52.7)**
**Dietary supplements**:	**115 (11.6)**	**464 (46.6)**
Glucosamine	< 1%	164 (16.5)
Herbal †	41 (4.1)	87 (8.7)
Minerals	45 (4.5)	129 (13.0)
Multivitamins	31 (3.1)	104 (10.4)
Omega-3	37 (3.7)	254 (25.5)
Vitamins	44 (4.4)	157 (15.8)
Other	18 (1.8)	85 (8.5)
**Non-medical treatments**: #	**17 (1.7)**	**84 (8.4)**
**Self help activities**:	**73 (7.3)**	**125 (12.6)**
Meditation	14 (1.4)	<5%
Prayer	56 (5.6)	77 (7.7)

Frequencies shown for CAM subcategories used by over 5% of men for any reason or by over 1% of men for prostate cancer and/or treatment side effects

^ Men may use more than one category or subcategory of CAM

^†^ Includes herbs and plant extracts

^#^ No single non-medical treatment subcategory was used by over 5% of men for any reason or by over 1% of men for prostate cancer.

Men were more likely to be CAM users for prostate cancer if they were regular or occasional participants in a support group (vs. no contact with support groups, RR = 2.02; 95%CI 1.41–2.88), born in another country (vs. Australian born RR = 1.59; 95%CI 1.17–2.16) or ever received androgen deprivation treatment (ADT) (RR = 1.60; 95%CI 1.12–2.28) (Table 2). CAM use for prostate cancer was also significantly higher among men who received ADT in the past two years (RR = 2.34; 95%CI 1.56–3.52) (Table 3).

**Table 2:** Associations between current CAM use for prostate cancer and/or its treatment side effects and socio-demographic/clinical characteristics for Australian long-term prostate cancer survivors.

Characteristic	Current CAM use for prostate cancer and/or treatment side effects (n = 996)				
	Non-user n (%)	User n (%)	Age-adjusted RR1 *	Fully-adjusted RR2 **	p-nominal p-trend^
	**828 (83.1)**	**168 (16.9)**			
**Age (years)**					
<65	132 (80.5)	32 (19.5)	ref.	ref.	0.240
65–69	223 (83.5)	44 (16.5)	0.84 (0.56, 1.28)	0.78 (0.50, 1.22)	0.080
70–74	245 (81.9)	54 (18.1)	0.93 (0.62, 1.37)	0.82 (0.52, 1.30)	
75–80	228 (85.7)	38 (14.3)	0.73 (0.48, 1.12)	0.63 (0.39, 1.01)	
**Education**					
University or college degree	237 (79.8)	60 (20.2)	ref.	ref.	0.260
High school	570 (85.1)	100 (14.9)	0.74 (0.55, 0.99)	0.90 (0.67, 1.21)	0.999
Less than high school	18 (72.0)	7 (28.0)	1.50 (0.76, 2.96)	1.56 (0.78, 3.09)	
Missing +	3 (75.0)	1 (25.0)			
**Socio-economic status of residence area**					
1- Highest SES	287 (82.9)	59 (17.1)	ref.	ref.	0.903
2	172 (84.3)	32 (15.7)	0.93 (0.63, 1.37)	0.86 (0.56, 1.31)	0.379
3	176 (82.2)	38 (17.8)	1.03 (0.71, 1.49)	0.87 (0.56, 1.34)	
4	121 (84.0)	23 (16.0)	0.94 (0.60, 1.46)	0.83 (0.49, 1.43)	
5- Lowest SES	70 (84.3)	13 (15.7)	0.92 (0.53, 1.60)	0.72 (0.35, 1.48)	
Missing +	2 (40.0)	3 (60.0)			
**Place of residence**					
Major city	480 (82.8)	100 (17.2)	ref.	ref.	0.419
Inner regional	226 (85.0)	40 (15.0)	0.87 (0.62, 1.22)	0.99 (0.68, 1.45)	0.195
Outer regional/ remote/ very remote	121 (81.8)	27 (18.2)	1.06 (0.72, 1.56)	1.33 (0.83, 2.14)	
Missing +	1 (50.0)	1 (50.0)			
**Health insurance**					
Private health insurance- with extras	477 (81.7)	107 (18.3)	ref.	ref.	0.195
Private health insurance- without extras	125 (81.7)	28 (18.3)	1.01 (0.69, 1.48)	1.00 (0.67, 1.50)	0.094
Medicare	224 (87.5)	32 (12.5)	0.69 (0.48, 0.99)	0.71 (0.49, 1.04)	
Missing +	2 (66.7)	1 (33.3)			
**Employment status**					
In full time paid work	110 (84.6)	20 (15.4)	ref.	ref.	0.386
In part time paid work	89 (83.2)	18 (16.8)	1.17 (0.64, 2.12)	1.32 (0.72, 2.44)	n/a
Retired/Unemployed	617 (83.4)	123 (16.6)	1.23 (0.76, 1.98)	1.23 (0.73, 2.07)	
Self-employed	10 (58.8)	7 (41.2)	2.84 (1.40, 5.76)	2.02 (0.90, 4.53)	
Missing +	2 (100.0)	0 (0.0)			
**Married or in defacto partner relationship**					
No	142 (85.5)	24 (14.5)	ref.	ref.	0.340
Yes	685 (82.6)	144 (17.4)	1.20 (0.80, 1.78)	1.23 (0.80, 1.89)	n/a
Missing +	1 (100.0)	0 (0.0)			
**Participate in a support group**					
No contact with support groups	655 (85.1)	115 (14.9)	ref.	ref.	<0.001
Receive newsletter only	124 (81.0)	29 (19.0)	1.27 (0.88, 1.84)	1.26 (0.85, 1.85)	n/a
Participate regularly or occasionally	49 (67.1)	24 (32.9)	2.20 (1.52, 3.17)	2.02 (1.41, 2.88)	
**Country of birth**					
In Australia	656 (85.4)	112 (14.6)	ref.	ref.	0.003
In another country	172 (75.8)	55 (24.2)	1.66 (1.24, 2.21)	1.59 (1.17, 2.16)	n/a
Missing +	0 (0.0)	1 (100.0)			
**Overall cancer severity at diagnosis**†					
Localised low risk	296 (86.8)	45 (13.2)	ref.	ref.	0.366
Localised intermediate risk	298 (83.0)	61 (17.0)	1.30 (0.91, 1.85)	1.21 (0.82, 1.79)	0.071
Localised high risk	136 (77.3)	40 (22.7)	1.76 (1.19, 2.58)	1.32 (0.85, 2.04)	
Stage T3-4	50 (73.5)	18 (26.5)	2.06 (1.27, 3.34)	1.62 (0.95, 2.75)	
Unknown	48 (92.3)	4 (7.7)	0.59 (0.22, 1.57)	0.69 (0.25, 1.88)	
**Treatments used since diagnosis** ^^					
Active surveillance	83 (83.8)	16 (16.2)	1.14 (0.69, 1.89)	1.32 (0.76, 2.29)	0.001 #
Prostatectomy	563 (85.6)	95 (14.4)	0.89 (0.61, 1.29)	0.76 (0.51, 1.14)	n/a
EBRT/Brachytherapy	310 (78.1)	87 (21.9)	1.12 (0.75, 1.66)	1.03 (0.68, 1.57)	
Bone EBRT	5 (83.3)	1 (16.7)	1.27 (0.26, 6.27)	0.81 (0.14, 4.51)	
Androgen deprivation treatment	237 (74.3)	82 (25.7)	1.92 (1.37, 2.67)	1.60 (1.12, 2.28)	
Chemotherapy/bisphosphonates +	7 (100.0)	0 (0.0)			

* Adjusted for age;

** Adjusted for age, education, socio-economic status of residence area, place of residence, health insurance, employment status, marital status, participation in a support group, country of birth, cancer severity at diagnosis, and treatments used since diagnosis;

^+^ Excluded from regression analyses;

^†^ Localised (stage 1 or 2) risk groups- low risk (PSA≤10, Gleason score ≤6, and clinical stage = T1-2a), intermediate risk (10<PSA≤20, Gleason score = 7 or clinical stage = T2b) high-risk (PSA >20, Gleason score>7, or clinical stage T2c); p-values values correspond to fully adjusted models;

^p-trend analysis excludes ‘missing’ and ‘unknown’ categories;

^^ Multiple treatments possible for each man and reference group for each treatment is not having had that treatment;

^#^ p-value is for test that all RR2s equal one; Data from 10-year survey unless “at diagnosis” stated.

**Table 3:** Associations between current CAM use for prostate cancer and/or its treatment side effects and other clinical characteristics for Australian long-term prostate cancer survivors.

Characteristic	Current CAM use for prostate cancer and/or treatment side effects (n = 996)				
	Non-user n (%)	User n (%)	Age-adjusted RR1 *	Fully-adjusted RR2 **	p-nominal p-trend ^
	**828 (83.1)**	**168 (16.9)**			
**PSA at diagnosis (ng/mL)** †					
<4	72 (84.7)	13 (15.3)	ref.	ref.	0.688
4 to <10	477 (84.1)	90 (15.9)	1.06 (0.62, 1.82)	1.04 (0.60, 1.79)	0.529
10 to <20	168 (84.0)	32 (16.0)	1.08 (0.59, 1.97)	0.99 (0.53, 1.85)	
20+	70 (72.2)	27 (27.8)	1.85 (1.01, 3.36)	1.29 (0.66, 2.49)	
Unknown	41 (87.2)	6 (12.8)	0.85 (0.34, 2.12)	1.67 (0.60, 4.66)	
**Gleason score at diagnosis** †					
<7	452 (85.3)	78 (14.7)	ref.	ref.	0.963
7	284 (80.5)	69 (19.5)	1.32 (0.98, 1.77)	1.01 (0.74, 1.39)	0.499
>7	63 (77.8)	18 (22.2)	1.53 (0.97, 2.42)	0.90 (0.53, 1.52)	
Unknown	29 (90.6)	3 (9.4)	0.62 (0.20, 1.89)	0.92 (0.36, 2.36)	
**Clinical stage at diagnosis** †					
T1a-c	426 (85.0)	75 (15.0)	ref.	ref.	0.372
T2a-c	321 (81.7)	72 (18.3)	1.24 (0.93, 1.67)	1.00 (0.73, 1.36)	0.303
T3a-c, T4a	50 (73.5)	18 (26.5)	1.81 (1.15, 2.83)	1.33 (0.80, 2.20)	
Unknown	31 (91.2)	3 (8.8)	0.59 (0.19, 1.78)	0.48 (0.16, 1.49)	
**Knowledge of cancer spread** †					
No	783 (84.6)	142 (15.4)	ref.	ref.	0.177
Yes	45 (63.4)	26 (36.6)	2.40 (1.71, 3.38)	1.34 (0.88, 2.05)	n/a
**Active treatments used in past 2 years** †					
None	750 (85.6)	126 (14.4)	ref.	ref.	<0.001
Androgen deprivation treatment	59 (60.8)	38 (39.2)	2.85 (2.12, 3.83)	2.34 (1.56, 3.52)	n/a
Other active treatments ^^	19 (82.6)	4 (17.4)	1.17 (0.48, 2.86)	1.01 (0.37, 2.73)	

* Adjusted for age;

** Adjusted for age, education, socio-economic status of residence area, place of residence, health insurance, employment status, marital status, participation in a support group, country of birth, PSA at diagnosis, Gleason score at diagnosis, clinical stage at diagnosis, knowledge of cancer spread and active treatments used in the past 2 years;

^†^ To avoid collinearity, PSA at diagnosis, Gleason score at diagnosis, clinical stage at diagnosis, knowledge of cancer spread and active treatments used in the past 2 years were not included in models simultaneously with overall cancer severity at diagnosis and treatments used since diagnosis; p-values values correspond to fully adjusted models.

^p-trend analysis excludes ‘missing’ and ‘unknown’ categories;

^^ Does not include men who received ADT in past 2 years; Data from 10-year survey unless “at diagnosis” stated.

While cancer severity at diagnosis was not associated with CAM use in the fully-adjusted model (p = 0.366; p-trend = 0.071) in Table 2, when ‘treatments used since diagnosis’ was removed from the model then higher risk prostate cancer at diagnosis became significantly associated with an increased likelihood of CAM use (p = 0.010; p-trend = 0.001). Similarly, while knowledge of cancer spread was not associated with CAM use in the fully-adjusted model (p = 0.177) in Table 3, when ‘treatments used in past 2 years’ was removed from the model then knowledge of cancer spread became significantly associated with an increased likelihood of CAM use (p<0.001).

Additional adjustment for selected individual psychological domains—fear of recurrence, cancer-specific distress, hyperarousal, cognitive avoidance and anxiety—did not substantively alter the estimated adjusted RRs and 95% CIs for the associations between CAM use for prostate cancer and patient characteristics (data available on request).

**Fig 1:**
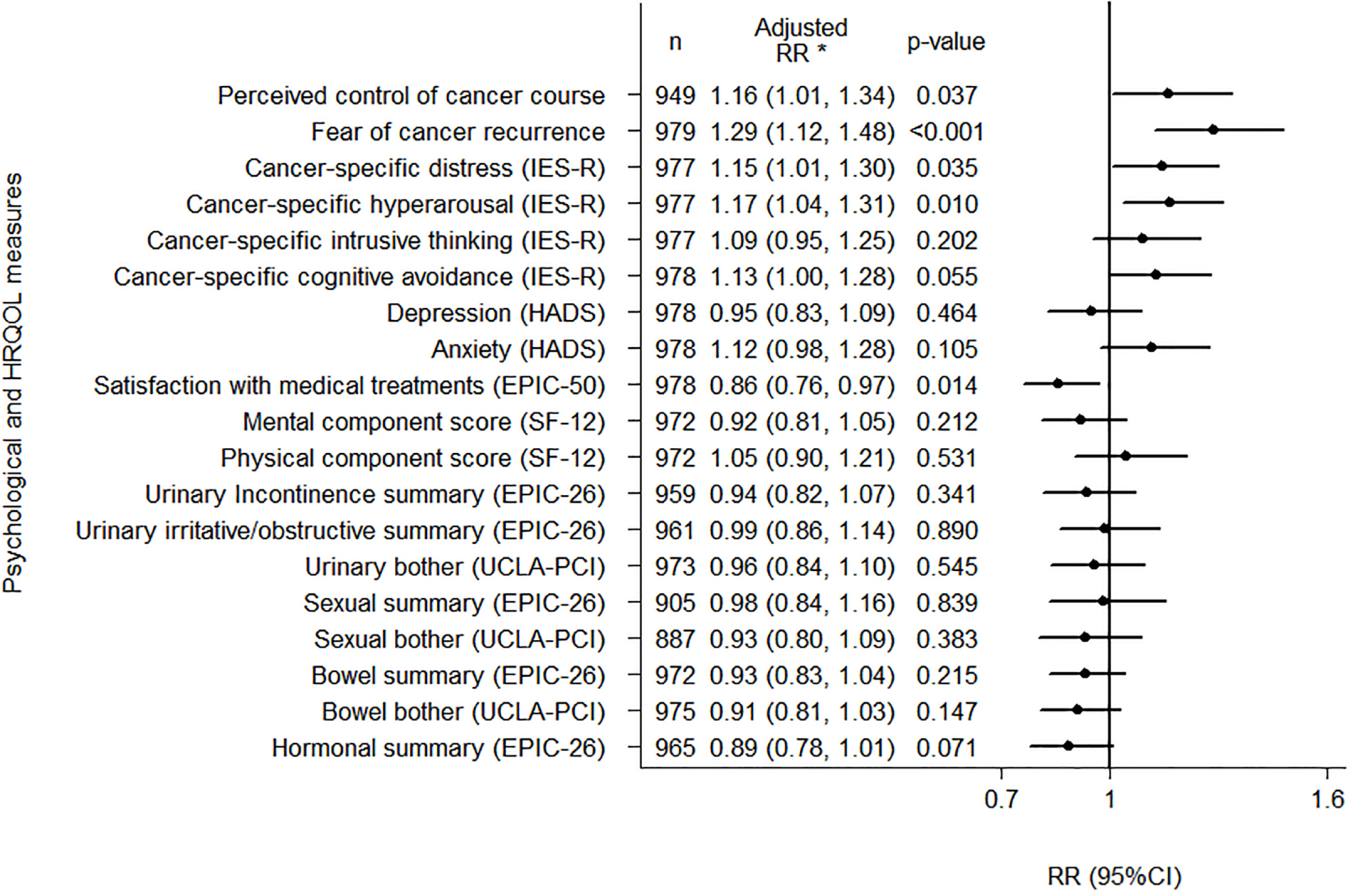
Relative risk (RR) of current CAM use for prostate cancer and/or its treatment side effects per standard deviation increase in psychological or HRQOL domain for Australian long-term prostate cancer survivors. *Adjusted for age, education, socio-economic status of residence area, place of residence, health insurance, employment status, marital status, participation in a support group, country of birth, cancer severity at diagnosis and treatments used since diagnosis.

For the psychological domains, CAM use for prostate cancer was associated with greater perceived control of cancer course (RR = 1.16; 95%CI 1.01–1.34), fear of recurrence (RR = 1.29; 95%CI 1.12–1.48), cancer-specific distress (RR = 1.15; 95%CI 1.01–1.30) and hyperarousal (RR = 1.17; 95%CI 1.04–1.31). CAM use for prostate cancer was more likely with less satisfaction with medical treatments (RR = 0.86; 95%CI 0.76–0.97), but was not associated with intrusive thinking, cognitive avoidance, depression, anxiety or any of the HRQOL domains (*p*-values ranged from 0.055 to 0.890) (Fig 1).

## Discussion

In this cohort of long-term prostate cancer survivors, just over half of men used CAMs for any reason and about one in six used them for prostate cancer. Men were more likely to use CAMs for prostate cancer if they were born in a country other than Australia, received ADT and/or were less satisfied with the medical treatments they received. A higher likelihood of CAM use for prostate cancer was associated with having greater cancer-related distress and/or having a greater fear of cancer recurrence, suggesting that psychological factors may play a part in driving this behaviour. CAM use was also more likely among men with a greater belief that their cancer course can be controlled as well as men who participated in support groups.

While there have been a number of studies that have quantitatively examined factors associated with CAM use among men diagnosed with prostate cancer, only seven of these studies specifically measured CAM use for prostate cancer [8–11,32–34]. We consider this distinction important, as the factors that motivate CAM use for prostate cancer are likely to differ from those that motivate CAM use for other purposes. Although none of the seven previous studies were restricted exclusively to long-term survivors, comparisons with the current study results are informative. Consistent with the findings of the current study, CAM use was not found to be associated with marital status in four previous studies to examine this association [8–10,32]. CAM use was found to be associated with younger age in three of six previous studies [8–11,32–33]. In the current study, although the lowest and highest proportions of CAM users were in the oldest and youngest age groups respectively (14% vs 20%), the association between age and CAM use was not statistically significant (p-trend = 0.080). While higher education has been found to be associated with CAM use in two of three previous studies [8–10], we found little evidence for such a relationship in an Australian setting. We also found that men who participated in prostate cancer support groups were more likely to be CAM users and this was in agreement with the two previous studies that examined this [8,10]. Because of the cross-sectional nature of these studies and ours, however, it is not clear whether the likelihood of CAM use becomes raised as a consequence of joining a support group or whether support group participants were predisposed to CAM use before joining.

The current study found that receiving ADT either at any time since diagnosis or in the past two years was associated with an increased likelihood of CAM use, and similar findings have been reported in one other study [10]. This is consistent with the view that recipients of ADT may use CAMs in an attempt to ameliorate the more severe side effects and risks associated with ADT in comparison to other treatments. Moreover, indicators of cancer severity and knowledge of cancer spread were not found to be associated with CAM use in the current study, except in the analyses not adjusted for treatments received. These results, taken together, suggest that the increased CAM use among patients with higher risk disease characteristics appears driven more by the hope of ameliorating ADT side effects than by the hope of reducing disease risks. This interpretation is supported by two previous studies [9,32] that also did not find an association between CAM use and disease severity, but is at odds with another study that found increased CAM use among patients with higher Gleason score at diagnosis [11].

We found that CAM use for prostate cancer was associated with higher levels of cancer-specific distress, cancer-specific hyperarousal and fear of cancer recurrence. It may be that for some men CAM use is part of an active response to psychological distress. Men are typically low users of traditional cancer support programs and researchers have suggested that men-centred support programs that tap into masculine values around self-reliance may be more acceptable and effective [35,36]. A high use of CAM in this population may reflect the ability of these products to tap into this dynamic more effectively than traditional services. Similar results have been reported in a study examining CAM use amongst early-stage breast cancer patients [37] where it was suggested that women may start using CAMs as a response to psychological symptoms or distress. Although our results suggest that this may also be true for long-term prostate cancer patients, it should be noted that this finding appears somewhat inconsistent with that of Steginga et al [33]. In that study, men using CAMs for prostate cancer were found to be less psychologically distressed than non-users at 12 months after their first treatment, although the number of users was small (n = 14).

In the present study, perceived control of the course of cancer was found to be associated with CAM use. This finding has been observed for people with other types of cancer [38] and is consistent with the view that CAM use might provide men with a sense of being active contributors to their own health and well-being. However, whether such beliefs are good for cancer patients is unclear. While optimism can have a positive impact on psychological health, optimism predicated on fallacy might ultimately lead to greater disappointment and psychological suffering.

The use of CAMs for prostate cancer was not found to be associated with HRQOL scores for any of the HRQOL domains examined in the current study. These findings are similar to those of three previous studies [9–10,32] which found no associations between CAM use and HRQOL for each of the UCLA-PCI bowel, sexual and urinary domains other than a weak association between CAM use and greater bowel bother (p = 0.035) [32]. Hence, the HRQOL of men with prostate cancer does not seem to be an important motivator of CAM use, nor does CAM use seem to be an important contributor to better or worse HRQOL.

This study has several limitations. First, the definition of CAMs encompasses a wide range of therapies and, consequently, the factors associated with individual CAMs may differ from the factors associated with all CAMs combined. Second, potential interactions between the factors associated with CAM use were not examined in this study because there was insufficient statistical power to do so effectively. Third, a potentially important source of bias is the non-response of men in the PCOS cohort to the 10-year survey. Somewhat reassuringly, however, respondents (representing 61% of living participants) and non-respondents had similar demographic and clinical characteristics (S2 Table), except for a higher proportion of non-responders in the oldest age group. Fourth, because CAM use was not measured in the PCOS cohort at the time of diagnosis, the associations reported in this study are cross-sectional in nature. Hence, the data do not allow us to ascribe causal links to our estimates of association or to make direct comparisons between the CAM use of recently diagnosed men and long-term survivors.

The findings of our study may be useful for doctors who care for long-term prostate cancer survivors. In particular, given that CAM use is associated with a number of psychological characteristics, the discovery of a patient’s CAM use may prompt discussion of the potential benefits of counselling. Furthermore, given that some men may be seeking to control the course of their cancer through CAMs, some users may be more amenable to doctors’ recommendations for therapies that are potentially beneficial, such as healthy lifestyle interventions.

## Supporting information

S1 Table Reasons for—and sources of information on—Current CT use for prostate cancer and/or its treatment side effects. (PDF)

S2 Table Demographic and clinical characteristics of PCOS men invited to participate in the 10-year survey; respondents vs. non-respondents. (PDF)

S3 Table Dietary supplements currently used for prostate cancer and/or treatment side effects and for any reason. (PDF)

S1 Fig Flow diagram showing patients’ participation and follow-up. (PDF)
