# Supplementary material for: Factors associated with the use of complementary and alternative medicines for prostate cancer by long-term survivors
Source: PLoS One. 2018 Mar 7;13(3):e0193686. doi: 10.1371/journal.pone.0193686 (PMC5841769; doi:10.1371/journal.pone.0193686)
Supplement: S1 Table — (PDF) [file pone.0193686.s001.pdf]

S1 Table. Reasons for ─ and sources of information on ─ current CT use for prostate cancer and/or its treatment side effects

	Dietary supplements n=115 n (%)	Self help activities n=73 n (%)
**Reasons for current use‡:**		
To boost my immune system	74 (64.4)	15 (20.6)
To prevent cancer returning or spreading	39 (33.9)	21 (28.8)
To deal with the side effects of treatment	21 (18.3)	7 (9.6)
To make me feel better	35 (30.4)	26 (35.6)
To cure the cancer	11 (9.6)	8 (11.0)
To cope with the stress	11 (9.6)	22 (30.1)
To provide hope	10 (8.7)	27 (37.0)
To do as much as I can for myself	38 (33.0)	31 (42.5)
Because they are not toxic	13 (11.3)	7 (9.6)
Because I am disappointed in the other treatments	1 (0.9)	0 (0)
Other	11 (9.6)	4 (5.5)
Not answered	1 (0.9)	4 (5.5)
**Sources of information on CTS‡:**		
Other men with prostate cancer	6 (5.2)	3 (4.1)
Friends or relatives	31 (27.0)	19 (26.0)
Newspaper, mags, radio TV	37 (32.2)	11 (15.1)
Internet	13 (11.3)	3 (4.1)
Doctor	37 (32.2)	5 (6.9)
Religious practice	0 (0)	20 (27.4)
Naturopath/herbalist	5 (4.4)	0 (0)
Books	3 (2.6)	1 (1.4)
Allied health	3 (2.6)	1 (1.4)
Other	2 (1.7)	9 (12.3)
Not answered	10 (8.7)	12 (16.4)

Non-medical treatments excluded due to low number (n=17)

‡ Multiple response question; percentages may add to more than 100%
