# Supplementary material for: Factors associated with the use of complementary and alternative medicines for prostate cancer by long-term survivors
Source: PLoS One. 2018 Mar 7;13(3):e0193686. doi: 10.1371/journal.pone.0193686 (PMC5841769; doi:10.1371/journal.pone.0193686)
Supplement: S2 Table — (PDF) [file pone.0193686.s002.pdf]

S2 Table. Demographic and clinical characteristics of PCOS men invited to participate in the 10-year survey; respondents vs. non-respondents

	PCOS 10-year survey	
**Characteristic**	**Respondents† n (%)**	**Non-respondents or PCOS men lost to follow-up† n (%)**
	**996 (100.0)**	**638 (100.0)**
**Age ^**		
<65	164 (16.5)	84 (13.2)
65-69	267 (26.8)	143 (22.4)
70-74	299 (30.0)	181 (28.4)
75-80	266 (26.7)	230 (36.1)
**Education**		
University or college degree	297 (29.8)	150 (23.5)
High school	670 (67.3)	446 (69.9)
Less than high school	25 (2.5)	41 (6.4)
Missing	4 (0.4)	1 (0.2)
**Socio-economic status of residence area at time of diagnosis**		
1- Highest SES	387 (38.9)	222 (34.8)
2	188 (18.9)	126 (19.7)
3	204 (20.5)	123 (19.3)
4	140 (14.1)	99 (15.5)
5- Lowest SES	73 (7.3)	66 (10.3)
Missing	4 (0.4)	2 (0.3)
**Place of residence at time of diagnosis**		
Major city	687 (69.0)	442 (69.3)
Inner regional	238 (23.9)	144 (22.6)
Outer regional/ remote/ very remote	68 (6.8)	50 (7.8)
Missing	3 (0.3)	2 (0.3)
**Country of birth**		
In Australia	768 (77.1)	464 (72.7)
In another country	227 (22.8)	173 (27.1)
Missing	1 (0.1)	1 (0.2)
**Overall cancer severity at diagnosis ^^**		
Localised low risk	341 (34.2)	197 (30.9)
Localised intermediate risk	359 (36.0)	191 (29.9)
Localised high risk	176 (17.7)	132 (20.7)
Stage T3-4	68 (6.8)	43 (6.7)
Missing	52 (5.2)	75 (11.8)
**First treatment after diagnosis**		
Active Surveillance	99 (9.9)	79 (12.4)
Androgen Deprivation Therapy	25 (2.5)	21 (3.3)
Combined EBRT/ADT	89 (8.9)	66 (10.3)
External Beam Radiotherapy	62 (6.2)	43 (6.7)
HDR Brachytherapy	48 (4.8)	11 (1.7)
LDR Brachytherapy	40 (4.0)	13 (2.0)
Orchidectomy	0 (0.0)	3 (0.5)
Radical Prostatectomy	608 (61.0)	349 (54.7)
Missing	25 (2.5)	53 (8.3)

† Respondents are men who completed the 10-year survey; Non-respondents are living men in the PCOS cohort remaining in the study as of January 2011, who were invited but did not complete the 10-year survey; PCOS men lost follow-up includes PCOS participants who, as of January 2011, had withdrawn from PCOS or were no longer contactable; ^ Age represents age at date of 10-year survey completion for respondents, and age at median date of 10-year survey completion for non-respondents and PCOS men lost follow-up;^^ Localised (stage 1 or 2) risk groups- low risk (PSA≤10, Gleason score ≤6, and clinical stage=T1-2a), intermediate risk (10<PSA≤20, Gleason score=7 or clinical stage=T2b) high-risk (PSA >20, Gleason score>7, or clinical stage T2c);
