# Supplementary material for: Factors associated with the use of complementary and alternative medicines for prostate cancer by long-term survivors
Source: PLoS One. 2018 Mar 7;13(3):e0193686. doi: 10.1371/journal.pone.0193686 (PMC5841769; doi:10.1371/journal.pone.0193686)
Supplement: S3 Table — (PDF) [file pone.0193686.s003.pdf]

S3 Table. Dietary supplements currently used for prostate cancer and/or treatment side effects and for any reason

Supplement ^	For prostate cancer and/or treatment side effects n (% of 996)	For any reason n (% of 996)
**Glucosamine**	<2%	**164 (16.5)**
**Minerals:**	**45 (4.5)**	**129 (13.0)**
calcium	42 (4.2)	139 (14.0)
copper	26 (2.6)	106 (10.6)
iodine	29 (2.9)	92 (9.2)
iron	20 (2.0)	79 (7.9)
magnesium	36 (3.6)	133 (13.4)
selenium	38 (3.8)	109 (10.9)
zinc	48 (4.8)	149 (15.0)
**Omega-3:**	**37 (3.7)**	**254 (25.5)**
fish oil †	26 (2.6)	196 (19.7)
**Herbal:***	**41 (4.1)**	**87 (8.7)**
bioflavonoids	<2%	50 (5.0)
**Multivitamins:**	**31 (3.1)**	**157 (15.8)**
betacarotene	24 (2.4)	65 (6.5)
folic acid	36 (3.6)	127 (12.8)
vitamin A	28 (2.8)	85 (8.5)
vitamin B1	34 (3.4)	130 (13.1)
vitamin B2	34 (3.4)	123 (12.4)
vitamin B3	34 (3.4)	122 (12.3)
vitamin B6	40 (4.0)	138 (13.9)
vitamin B12	38 (3.8)	137 (13.8)
vitamin C	45 (4.5)	180 (18.1)
vitamin D	51 (5.1)	151 (15.2)
vitamin E	46 (4.6)	137 (13.8)

Frequencies shown for supplements used by over 5% of participants for any reason or by over 2 % of participants for prostate cancer and/or treatment side effects

^ Participants may use more than one supplement

† including omega 3 triglyceride concentrates

* Includes herbs and plant extracts
