# Supplementary figures and images for: Factors associated with the use of complementary and alternative medicines for prostate cancer by long-term survivors
Source: PLoS One. 2018 Mar 7;13(3):e0193686. doi: 10.1371/journal.pone.0193686 (PMC5841769; doi:10.1371/journal.pone.0193686)

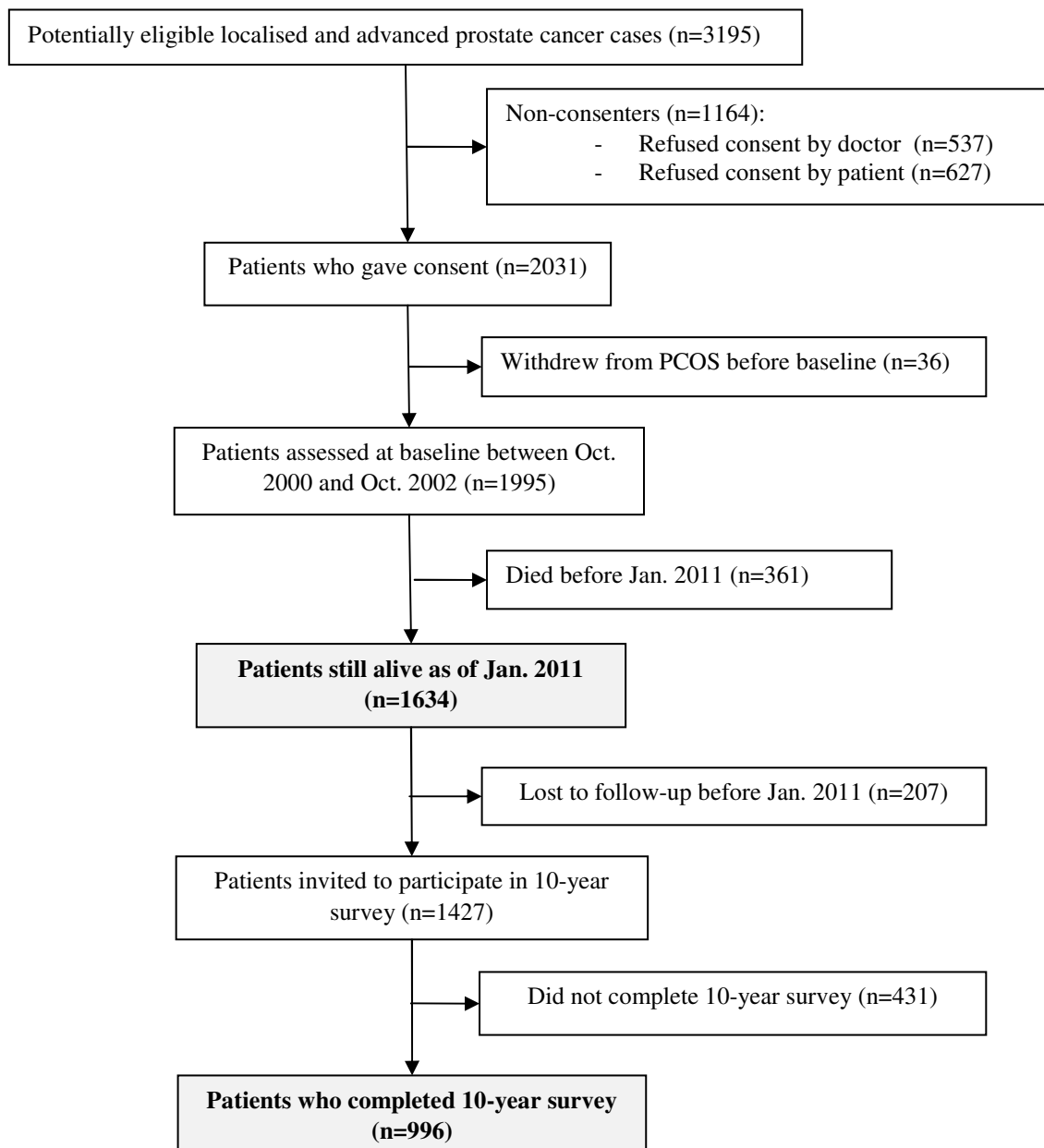

**S1 Fig. Flow diagram showing patients' participation and follow-up**

Supplement: S1 Fig — (PDF) [file pone.0193686.s004.pdf]
